# Optimization of the Fermentation Conditions of Huaniu Apple Cider and Quantification of Volatile Compounds Using HS-SPME-GC/MS

**DOI:** 10.3390/metabo13090998

**Published:** 2023-09-08

**Authors:** Yuwen Mu, Chaozhen Zeng, Ran Qiu, Jianbin Yang, Haiyan Zhang, Juan Song, Jing Yuan, Jing Sun, Sanjiang Kang

**Affiliations:** 1 Agricultural Product Storage and Processing Research Institute, Gansu Academy of Agricultural Sciences, Lanzhou 730070, China; muyw@gsagr.cn (Y.M.); zengchaozhen@gsagr.cn (C.Z.); zhanghaiyan@gsagr.cn (H.Z.); songjuan@gsagr.cn (J.S.); yuanjing@gsagr.cn (J.Y.); 2 Key Laboratory of Agro-Products Processing, Ministry of Agriculture and Rural Affairs, Institute of Food Science and Technology, Chinese Academy of Agricultural Sciences, Beijing 100193, China; caasmu23@gmail.com; 3 China Resources Beer (Holdings) Company Limited, Beijing 100005, China; qiuran@crb.cn

**Keywords:** Huaniu apple, cider, RSM, volatile compounds, HS-SPME-GC/MS

## Abstract

The fermentation process and composition of volatile compounds play a crucial role in the production of Huaniu apple cider. This study aimed to optimize the fermentation conditions of Huaniu apple cider and quantify its volatile compounds using headspace solid-phase microextraction-gas chromatography–mass spectrometry (HS-SPME-GC/MS). The optimal fermentation parameters were determined using response surface methodology (RSM). The optimal fermentation temperature was 25.48 °C, initial soluble solids were 18.90 degrees Brix, inoculation amount was 8.23%, and initial pH was 3.93. The fermentation rate was determined to be 3.0, and the predicted value from the verification test was 3.014. This finding demonstrated the excellent predictability of a RSM-optimized fermentation test for Huaniu apple cider, indicating the reliability of the process conditions. Moreover, the analysis of volatile compounds in the optimized Huaniu cider identified 72 different ingredients, including 41 esters, 16 alcohols, 6 acids, and 9 other substances. Notably, the esters exhibited high levels of ethyl acetate, ethyl octanoate, and ethyl capricate. Similarly, the alcohols demonstrated higher levels of 3-methyl-1-butanol, phenethylethanol, and 2-methyl-1-propanol, while the acids displayed increased concentrations of acetic acid, caproic acid, and caprylic acid. This study provides the essential technical parameters required for the preparation of Huaniu apple cider while also serving as a valuable reference for investigating its distinct flavor profile.

## 1. Introduction

Huaniu apples are a series of cultivars derived from Red Delicious apples (*Malus domestica*, cv. Red Delicious) and cultivated in the province of Gansu, China. Renowned as a distinguished apple brand, Huaniu apples are comparable to American Red Delicious apples and Japanese Fuji apples [1]. They are primarily cultivated in Tianshui City, situated in Gansu Province, China, and have gained recognition as a distinctive local agricultural product. The unique flavor, exceptional taste, and high-quality attributes of Huaniu apples can be attributed to the specific production environment found in Tianshui [2]. However, these apples have a relatively short storage period, are prone to sponginess, and face challenges such as oversupply caused by the expansion of cultivation areas and a downturn in the international market [3,4]. The overreliance on fresh consumption leads to significant price fluctuations, thereby hindering the development of the Huaniu apple industry. Consequently, there is an urgent need for diversifying the processing of Huaniu apples.

Currently, apple diversification processing includes various products such as cider, vinegar, brandy, and slices, in addition to fresh fruits [5,6,7,8]. Cider, in particular, has experienced rapid growth, with the global cider industry’s value exceeding USD 500 million in 2020 [9]. The characteristics of cider depend on the apple variety and the stage of maturity of the raw material. Different apple varieties exhibit distinct aroma and flavor characteristics due to variations in soluble sugars, organic acids, volatile compounds, and other components [10]. The quality of cider products is closely linked to the inherent characteristics of the apple varieties. The quality criteria for apple processing exhibit notable distinctions compared to the conventional cultivation practices intended for fresh consumption. Factors such as sugar/acid ratio, polyphenols, and antioxidant capacity play crucial roles. In particular, phenolic compounds in apples participate in fermentation, contribute to the aroma of the fermentation juice, prevent microbial spoilage, and control the fermentation rate [11,12]. It has been reported that the concentration of polyphenols varies significantly among different apple varieties, with total phenolic content ranging from 107 mg/kg FW to 941 mg/kg FW [13,14].

Aroma is a vital indicator of cider quality and contributes to its distinctive flavor profile. The aroma of cider primarily consists of esters, alcohols, fatty acids, aldehydes, ketones, and terpenes. Recent research on cider aroma mainly focuses on enhancing it through the optimization of fermentation strains and processes. However, there is a relative lack of research concerning the variations in cider aroma among different apple varieties. With its distinct flavor and nutritional value, the Huaniu apple holds great promise for making fruit wine. However, we currently have limited knowledge about how Huaniu apple cider is fermented and what gives it its unique taste. Therefore, the primary objective of this study is to investigate the distinct flavor components of Huaniu apple cider by optimizing the fermentation process. By doing so, the aim of this study is to improve both the production process and the quality of the final product. This research will emphasize the significance of Huaniu apple cider in the context of industrial development, showcasing its potential and value in the market.

## 2. Materials and Methods

### 2.1. Cider Production

*Saccharomyces cerevisiae* 1023, obtained from the China Center of Industrial Culture Collection (CICC), was used for the fermentation process. Huaniu apples were harvested in Tianshui City, Gansu Province in 2021 and stored at 4 °C after harvesting. *Saccharomyces cerevisiae* preserved in a beveled medium was inoculated into a 250 mL triangular flask containing 100 mL of liquid seed medium and incubated at 28 °C for 48 h. The activated *Saccharomyces cerevisiae* was then inoculated into a triangular flask containing 500 mL of fermented apple juice at a different inoculation amount with a concentration of 10^6^ cfu/mL. The fermentation process was carried out anaerobically at different fermentation temperatures, initial total soluble solids, and initial pH following RSM until a constant weight was reached.

### 2.2. Total Soluble Solid Content and pH Measurement

The measurement of total soluble solids (TSS) content was conducted using a handheld refractometer. Similarly, the pH level was determined utilizing a pH meter.

### 2.3. Fermentation Rate


$$Fermentation\;Rate=\frac{\left(Weight_{\;before\;fermentation}-Weight_{\;after\;fermentation}\right)}{\left(TSS_{\;before\;fermentation}-TSS_{\;after\;fermentation}\right)}$$

### 2.4. Analysis of Cider Aroma Composition

Extraction of cider aroma components: 5 mL of the sample was added to 1 g of sodium chloride and 50 μL of internal standard 3-octanol. The mixture was loaded into a 15 mL headspace bottle. The vials were processed in the TriPlus RSH Autosampler-SPME system using fibers with a composition of 50/30 μm DVB/CAR/PDMS. The extraction conditions included an equilibration temperature of 60.0 °C for 30 min and an adsorption time of 30 min.

GC-MS conditions: The chromatographic conditions included an injection port temperature of 250 °C, desorption for 3 min, no split mode, and a carrier gas flow rate of 1.2 mL/min. The column used was DB-WAX (30 m × 0.25 mm × 0.25 μm). The heating procedure involved maintaining a constant temperature of 40 °C for 3 min, followed by an increase to 180 °C at a rate of 6 °C/min for 2 min, and finally raising the temperature to 230 °C at a rate of 10 °C/min for 6 min. The mass spectrometry conditions consisted of an EI ion source with an electron energy of 70 eV, an ion source temperature of 200 °C, and an interface temperature of 230 °C. The scan range was set from 33.00 to 450.00 amu.

### 2.5. Experimental Design and Data Analysis

Design-Expert software version 12.0 was used to optimize the fermentation temperature, initial soluble solids, inoculation amount, and initial pH for the fermentation rate (Table 1). All samples were tested in triplicate, and the results were reported as means ± standard deviation. The analysis of variance (ANOVA) was conducted using SPSS statistical software (version 23, SPSS Inc., Chicago, IL, USA), and differences were considered significant at *p* < 0.05.

## 3. Results and Discussion

### 3.1. Validation of the Experimental Design

Based on previous research, it has been identified that four factors, namely fermentation temperature, initial soluble solids, inoculum amount, and initial pH, have a significant impact on the fermentation of Huaniu apple cider. These factors have been carefully screened and found to play a crucial role in the overall fermentation process and the quality of the cider. Using Design Expert 12.0 software, a response surface analysis test was designed with 29 points, considering the four factors at three levels. The fermentation rate was chosen as the response value (Table 2). The fermentation rate ranged from 2.29 to 2.94.

The regression model for the fermentation process parameters of Huaniu apple cider was established. The fermentation rate was used as the response value, and the independent variables were fermentation temperature (X_1_), initial soluble solids (X_2_), inoculum amount (X_3_), and initial pH (X_4_).

$$\mathrm{Fermentation}\;\mathrm{Rate}\;R=2.54+0.14X_{2}+0.081X_{4}+0.11X_{24}+0.087X_{11}+0.077X_{22}-0.11X_{44}$$

The model was analyzed for variance, and the significance of the model coefficients was tested, taking the fermentation rate as the evaluation index (Table 3). The regression model was statistically significant (*p* < 0.001), indicating its significance. The initial soluble solids content had a significant effect on the fermentation rate (*p* < 0.001), and the initial pH had a significant effect as well (*p* < 0.01). The lack-of-fit term (*p* = 0.0692) was not significant, suggesting a good fit of the experimental model and reasonable model selection. The R^2^ value of the model was 0.8693, indicating a good fit between the measured and predicted values of Huaniu apple cider’s fermentation rate. The accuracy of the test (C.V.) was 3.13%, demonstrating the reliability of the experimental procedure.

### 3.2. Analysis of Response Surface

**Figure 1:**
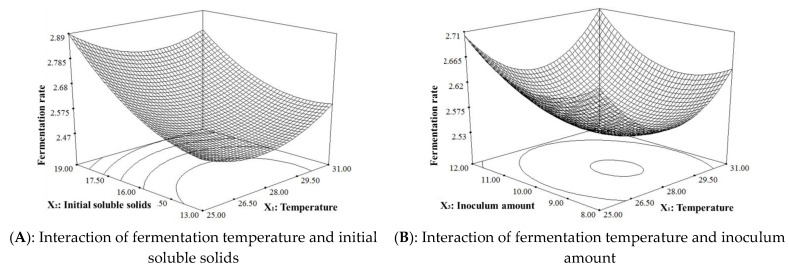

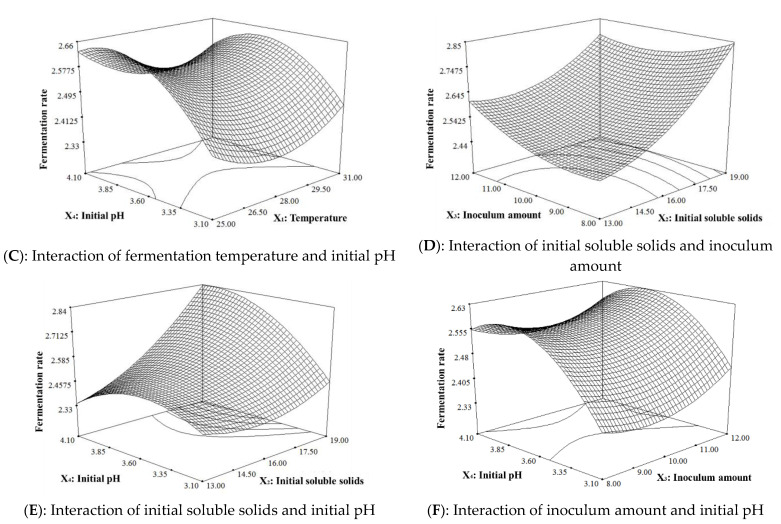
Response surface 3-D plots for the interaction effects of independent variables of temperature, initial soluble solids, inoculum amount, and initial pH on dependent variables of fermentation rate.

The 3-D response surface analysis (Figure 1) revealed that the fermentation rate of Huaniu apple cider increased with an increase in the initial soluble solids content. Fermentation rate refers to the process’s speed, often measured by product generation (like alcohol) or substrate consumption (such as sugars) over a set time. Similar to a speedometer, it mirrors car speed comprehension. In wine, beer, or yogurt production, precise pacing is vital for desired attributes. Slow pace extends timelines, and excessive speed alters traits. Controlling rate fine-tunes variables, enabling innovation and enhancement while ensuring process consistency. The initial soluble solids content is an important parameter in the cider fermentation process. A higher initial soluble solids content provides more fermentable sugars, resulting in a higher alcohol content [15]. Hence, the selection of the initial soluble solids content needs to strike a balance between the desired alcohol content and fermentation rate [16].

The fermentation temperature decreased the fermentation rate and then gradually increased it. The fermentation temperature is one of the key factors that affect the fermentation process and quality of cider. A higher fermentation temperature can promote yeast growth and metabolic activity, thereby speeding up fermentation [17]. However, excessively high temperatures may cause the yeast to produce an excessive number of metabolites that can affect the flavor of the cider [18]. Determining the appropriate fermentation temperature depends on the yeast strain used and the desired characteristics of the final product [19]. It is important to carefully control the production of yeast metabolites while maintaining an optimal fermentation rate.

The fermentation rate initially increased and then decreased with an increase in the initial pH. A suitable initial pH creates an environment conducive to yeast growth and metabolism. Higher pH values may inhibit yeast growth and fermentation activity, which can also affect the fermentation speed and product quality [20]. On the other hand, a lower pH may promote yeast growth but can also result in excessive acidity [21]. Therefore, selecting the appropriate initial pH requires considering the requirements of the yeast strain, the desired characteristics of the final product and maintaining the acid-base balance during fermentation.

The fermentation rate increased with an increase in the inoculum amount. The inoculation of *Saccharomyces cerevisiae* significantly affects the fermentation rate and product production of cider. Higher inoculum amounts generally accelerate fermentation because a larger number of yeast cells allows for faster conversion of sugars into alcohol and other metabolites. However, excessively high inoculation levels may lead to an accumulation of yeast metabolites, which can impact the flavor properties of the cider [22].

The interaction between the initial soluble solids and initial pH had a significant impact on the model. These provide valuable insights into the effects of fermentation initial soluble solids, *Saccharomyces cerevisiae* inoculation, fermentation temperature, and initial pH on the cider fermentation process. However, it is important to acknowledge that the specific influencing mechanism can be influenced by the interaction of multiple factors. Therefore, further research and optimization experiments are necessary in practical applications to determine the optimal combination of process parameters. The optimal fermentation process for Huaniu apple cider was determined through analysis using RSM. The determined optimal conditions were a fermentation temperature of 25.48 °C, initial soluble solids of 18.9 degrees Brix, inoculum amount of 8.23%, and initial pH of 3.93. The RSM helps save time and resources by finding the optimal combination of process parameters with fewer experiments. However, it is crucial to note that the RSM relies on certain assumptions and limitations, and the accuracy of the model depends on the quality of the experimental design and data acquisition. Therefore, in practical applications, it is necessary to integrate statistical methods and practical experience for process optimization and adjustment. To simplify the experimental operation, the modified extraction process conditions were adjusted to a fermentation temperature of 25.5 °C, initial soluble solids content of 18.9 degrees Brix, inoculum amount of 8.2%, and initial pH of 3.90. The fermentation rate was verified to be 3.0 (predicted value was 3.014) through a verification test, confirming the good predictability of the response-surface-optimized fermentation test for Huaniu apple cider. These results indicate the reliability of the fermentation conditions for this process.

### 3.3. TSS and pH

**Figure 2:**
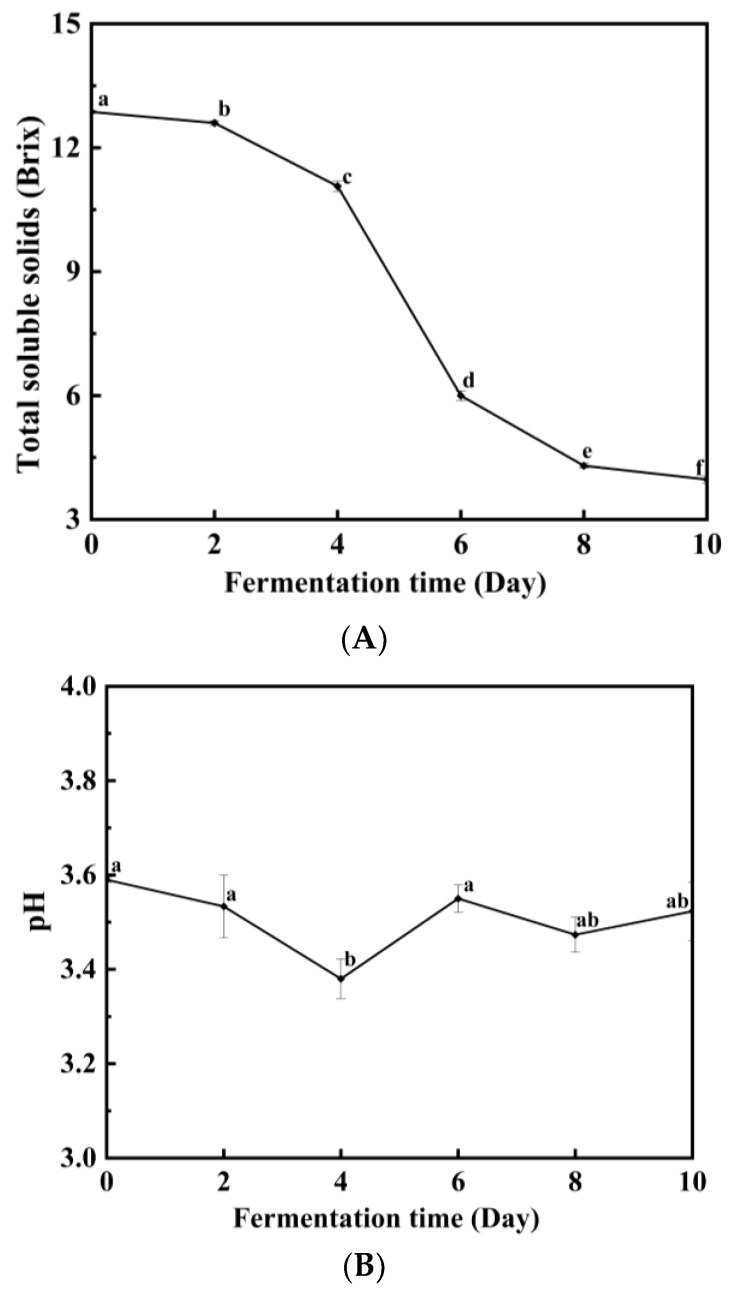
Changes of TSS (**A**) and pH (**B**) during fermentation. The different lowercase letters indicate significant differences (*p* < 0.05).

Figure 2 illustrates the changes in TSS and pH during the fermentation of Huaniu apple cider. The TSS content plays a significant role in the apple fermentation process. TSS during cider fermentation refers to the content of total solids that can be dissolved in cider, mainly composed of sugars, organic acids, amino acids, esters, and other dissolved substances in apple juice. The changes in soluble solids during cider fermentation are influenced by the metabolic activity and material transformation of yeast. As depicted in Figure 2A, the consumption of TSS content is relatively slow during the initial 4 days of fermentation, which can be attributed to the time required for the yeast to adapt to the fermentation environment of Huaniu apple juice. From day 4 to day 8, there is a rapid decrease in the TSS content, followed by a slower decrease or even stabilization from day 8 onwards. This pattern is related to the growth cycle and fermentation performance of the yeast. By the end of the 10-day fermentation period, the TSS content is reduced from the initial soluble solid 12.9 degrees Brix to 4.0 degrees Brix. The initial soluble solids content in the juice at the beginning of the cider fermentation process is an important factor in determining the sweetness and taste of the final product. It is usually measured in degrees Brix and represents the percentage of soluble solid substances based on glucose. During fermentation, yeast uses sugars from the fruit juice as substrates for fermentation. Yeast converts sugars into alcohol and carbon dioxide, resulting in a decrease in the soluble solids content in the fruit juice. As fermentation progresses, the concentration of sugars gradually decreases. During fermentation, organic acids, esters, and other metabolites produced by yeast gradually accumulate. These substances play a crucial role in the flavor and mouthfeel of cider. Their presence increases the complexity of cider and interacts with sugars, alcohol, and other components, affecting the soluble solids content and overall taste of cider [23]. At the end of fermentation, yeast converts most of the sugars into alcohol and other metabolites, resulting in a lower soluble solids content in cider. However, there may still be some residual sugar in cider, i.e., sugars that have not been completely converted by yeast, which will have an impact on the sweetness and taste of cider. It should be noted that the change in the soluble solids content of cider is affected by several factors, including the sugar/acid ratio of the raw fruit juice, the characteristics of the yeast strain, the fermentation temperature, the fermentation time, and more [24]. To control the change in soluble solids, adjustments can be made by modifying the amount of sugar added during fermentation and controlling the fermentation temperature and time, among other factors. This helps achieve the desired sweetness and taste while ensuring the quality and stability of the cider.

pH is a critical factor in the fermentation process as it profoundly influences the quality attributes of the yeast and the final product, such as flavor, color, and aroma. The results presented in Figure 2B demonstrate that the pH of Huaniu apple cider fluctuates within a narrow range during fermentation and tends to stabilize within a smaller range after the completion of alcoholic fermentation. The pH initially exhibits a slight decrease in the first 4 days and then a slight increase in the last 4 days. However, the final pH falls within the suitable range for cider production, indicating that the yeast has adapted and influenced the composition of the apple juice. The pH value of cider fermentation undergoes a series of changes due to the accumulation of organic acids and other compounds produced by yeast metabolism during the fermentation process, as well as the growth and metabolic activities of yeast. At the beginning of the fermentation process, the initial pH of the apple juice typically falls between 3.0 and 4.0. During the early stages of fermentation, the yeast initiates growth and utilizes sugars as the main metabolic substrate. The accumulation of organic acids gradually reduces the pH value of the fermentation liquid. In the middle of fermentation, the yeast’s metabolic activity reaches its peak. At this stage, the production of organic acids is high, resulting in a further decrease in the pH value of the fermentation liquid. As fermentation progresses, sugars are gradually converted by the yeast into alcohol and other metabolites, and the accumulation of organic acids is also reduced. This results in a gradual increase in the pH of the fermentation solution. The pH change in the cider fermentation process is influenced by various factors, including the acidity of the raw materials, the characteristics of yeast strains, fermentation temperature, and more [25,26]. Additionally, pH control in cider can be achieved by adjusting the initial pH and adding acidic or alkaline regulators. Ensuring the appropriate pH range helps promote yeast growth and metabolic activity while maintaining the quality and stability of the cider [27].

### 3.4. Volatile Compounds

The analysis of volatile components in the optimized Huaniu cider revealed a total of 72 main aroma compounds, including 41 esters, 16 alcohols, 6 acids, and 9 other substances (Table 4).

#### 3.4.1. Esters

Esters play a crucial role in imparting fruity and floral aromas to cider, significantly contributing to its overall flavor profile. In this study, 41 esters were detected, with higher levels of ethyl acetate, ethyl octanoate, ethyl caprate, ethyl caproate, and isoamyl acetate. Esters are formed through the reaction of alcohol with acetyl-CoA, catalyzed by acetyltransferase during yeast fermentation, as well as transesterification [28]. The formation of esters is influenced by various factors such as fermentation temperature, pH, nitrogen levels, microbial presence during fermentation, and growth-stimulating factors [29]. Ethyl acetate is the most abundant ester and is known to contribute significantly to the fruity aroma of cider when present at appropriate concentrations [30]. In the analyzed Huaniu cider samples, ethyl acetate was the predominant ester, accounting for over 25% of all volatile esters. Isoamyl acetate, another important ester, was also detected at relatively high concentrations, contributing to the pleasant fruity taste of Huaniu cider. Other esters, such as ethyl caprylate (with floral and fruity rose and orange aromas) and ethyl capricate (with a coconut aroma), play a role in giving cider its characteristic apple aroma [30].

#### 3.4.2. Alcohols

Alcohols are considered to have a significant impact on the aroma of cider. Higher concentrations of alcohols can result in strong pungent odors and flavors, while moderate amounts contribute to the characteristic fruit aroma [31]. In this study, a total of 16 alcohol compounds were detected, with higher levels of 3-methyl-1-butanol, phenethyl alcohol, 2-methyl-1-propanol, and 1-hexanol. Studies have shown that 3-methyl-1-butanol has aromas of cheese, phenethyl alcohol has a rose aroma, and 1-hexanol has flavors of grass and toast. The presence of these compounds in Huaniu cider may contribute to a more complex and layered aroma. Higher alcohols typically make up 0.1–0.7% of ethanol production, and excessive amounts can lead to undesirable odors and flavors. Appropriate concentrations of higher alcohols are crucial for providing the characteristic flavor of the fruit [32,33]. These alcohols are formed through the decarboxylation and deamination of corresponding amino acids, such as leucine, isoleucine, and valine, which influence the flavor of cider and contribute to its overall quality [34].

#### 3.4.3. Acids

Acids in cider primarily originate from fruit juice, acting as precursors of esters that enhance the pleasant aroma of cider. Moreover, these organic acids possess antiseptic properties, contributing to the physical and chemical stability of cider [35]. Maintaining an appropriate level of organic acids is crucial for achieving a balanced aroma in Huaniu cider. This study identified six organic acids with relatively high concentrations. Alongside acetic acid, which is the primary acid found in cider, caprylic acid and caproic acid are also prominent. The former imparts a sour scent reminiscent of butter or almonds, while the latter yields leafy, woody, and varnish notes. The content of organic acids significantly influences the flavor balance, taste, color, chemical stability, pH, nutritional properties, acceptability, and storage quality of cider [35,36].

#### 3.4.4. Others

In addition to esters and alcohols, the composition of cider is also influenced by the phenolic compounds present in apples [37,38]. Phenolic acids and flavan-3-ols are two important phenolic substances associated with apples and cider. Phenolic acids and flavan-3-ols contribute to the bitterness, astringency, color, and certain aromatic compounds in cider. Apples contain a variety of phenolic compounds, with significant variations between apple varieties [38,39]. Terpenes are aliphatic long-chain hydrocarbons derived from isoprene or dimethylallyl pyrophosphate, forming monoterpenes, sesquiterpenes, or diterpenes. They include oxidized derivatives such as alcohols, aldehydes, ketones, esters, and oxides. Previous studies have identified à-Farnesene, eugenol, piperonol, and isoeugenol in apple-fermented beverages [40,41,42].

Volatile esters, alcohols, aldehydes, and other compounds collaboratively shape the aroma profile of cider, significantly influencing its aroma characteristics and overall quality. When selecting apple varieties for juicing, it is important to prioritize those with high organoleptic and health properties, including good eating quality, rich flavor, and appealing juice appearance. Achieving optimal taste in single-variety apple juice relies on soluble solid measurement, with acidity acting as a vital regulator. Polyphenolic compounds contribute to the flavor and antioxidant activity of apple juice. With strong antioxidant properties attributed to its polyphenols and ascorbic acid, apple juice exceeds the total antioxidant activity of quercetin, epicatechin, and proanthocyanidin B2 in terms of vitamin C equivalents. Alcohols, aldehydes, and other volatile esters are the predominant volatile substances in cider, collectively shaping its aroma profile. 

## 4. Conclusions

This study aims to optimize the cider fermentation process using response surface optimization, enhancing product quality and taste. Through experiment design and response surface modeling, the optimal combination of process parameters was determined, leading to significant improvements in alcohol content, acidity, residual sugar, and flavor characteristics of the cider. The experimental results confirm the feasibility and stability of the optimized process parameters. In this study, we optimized the fermentation conditions for Huaniu apple cider, setting the temperature at 25.50 °C, initial soluble solid content at 18.90 degrees Brix, inoculum amount at 8.2%, and initial pH at 3.9. The extensive analysis conducted on the aroma components present in the optimized Huaniu cider has revealed a diverse collection of 72 distinct compounds. This comprehensive range prominently featured a significant abundance of esters, including ethyl acetate, ethyl octanoate, ethyl caprate, ethyl caproate, and isoamyl acetate, all of which were present at heightened concentrations. Furthermore, this characterization highlighted substantial levels of alcohols, with compounds such as 3-methyl-1-butanol, phenylethanol, 2-methyl-1-propanol, and 1-hexanol standing out. Particularly noteworthy was the augmentation in the presence of acids, with acetic acid, caproic acid, and caprylic acid playing a prominent role within the aromatic profile. These findings provide valuable insights into the preparation parameters and theoretical foundation for Huaniu apple cider, significantly contributing to the assessment of its aroma characteristics and overall quality.

## Figures and Tables

**Table 1:** Coded levels of independent variables for the Box–Behnken design (BBD).

Symbol	Independents	Coded Level		
		−α(−1)	0	+α(+1)
X_1_	temperature/°C	25	28	31
X_2_	initial soluble solids/°Brix	13	16	19
X_3_	inoculum amount/%	8	10	12
X_4_	initial pH	3.1	3.6	4.1

**Table 2:** BBD with responses of the dependent variables to fermentation rate.

Run	X_1_	X_2_	X_3_	X_4_	R (Predicted): Fermentation Rate	R (Actual): Fermentation Rate
1	25	13	10	3.6	2.53	2.62
2	31	13	10	3.6	2.60	2.51
3	25	19	10	3.6	2.89	2.93
4	31	19	10	3.6	2.80	2.67
5	28	16	8	3.1	2.36	2.29
6	28	16	12	3.1	2.44	2.41
7	28	16	8	4.1	2.56	2.54
8	28	16	12	4.1	2.57	2.60
9	25	16	10	3.1	2.42	2.41
10	31	16	10	3.1	2.46	2.50
11	25	16	10	4.1	2.63	2.60
12	31	16	10	4.1	2.57	2.59
13	28	13	8	3.6	2.45	2.46
14	28	19	8	3.6	2.84	2.94
15	28	13	12	3.6	2.61	2.53
16	28	19	12	3.6	2.78	2.79
17	25	16	8	3.6	2.67	2.60
18	31	16	8	3.6	2.65	2.70
19	25	16	12	3.6	2.70	2.68
20	31	16	12	3.6	2.71	2.81
21	28	13	10	3.1	2.39	2.45
22	28	19	10	3.1	2.46	2.47
23	28	13	10	4.1	2.34	2.36
24	28	19	10	4.1	2.84	2.81
25	28	16	10	3.6	2.54	2.61
26	28	16	10	3.6	2.54	2.51
27	28	16	10	3.6	2.54	2.51
28	28	16	10	3.6	2.54	2.55
29	28	16	10	3.6	2.54	2.53

**Table 3:** Model summary and ANOVA of fermentation rate.

Source	*F* Value	*p*-Value
Model	6.65	0.0005 ***
X_1_	0.046	0.8334
X_2_	36	0.0001 ***
X_3_	1.07	0.3179
X_4_	12	0.0038 **
X_12_	0.86	0.3692
X_13_	0.034	0.8554
X_14_	0.38	0.5461
X_23_	1.85	0.1951
X_24_	7.07	0.0187 *
X_34_	0.14	0.7161
X_11_	7.5	0.0160 *
X_22_	5.87	0.0295 *
X_33_	2.81	0.1161
X_44_	12.42	0.0034 **
Lack of fit		0.0692
R^2^		0.8693
C.V./%		3.13

X_1_ = temperature (°C), X_2_ = initial soluble solids (°Brix), X_3_ = inoculum amount (%), and X_4_ = initial pH; level of significance * *p* < 0.05, ** *p* < 0.01, and *** *p* < 0.001.

**Table 4:** Concentrations of volatiles (μg/L) in Huaniu apple cider.

Compound	Content (μg/L)	Compound	Content (μg/L)
Esters		2-Ethylhexyl acetate	0.86 ± 0.01
Ethyl Acetate	4198.21 ± 43.56	Ethyl 3-nonenoate	0.72 ± 0.02
Ethyl octanoate	991.21 ± 18.27	Ethyl 4-decenoate	0.63 ± 0.01
Ethyl decanoate	695.52 ± 12.33	Alcohols	
Ethyl caproate	131.65 ± 8.71	Ethanol	3023.72 ± 28.90
Isoamyl acetate	114.67 ± 8.33	3-Methyl-1-butanol	1156.91 ± 19.86
Acetic acid—2-phenyl ethyl ester	94.66 ± 5.15	Phenethyl ethanol	390.82 ± 15.21
Ethyl 9-decenoate	71.20 ± 5.28	2-Methyl-1-propanol	233.78 ± 9.98
Ethyl nonanoate	61.47 ± 4.94	1-Hexanol	208.89 ± 10.37
Ethyl lactate	45.27 ± 1.08	3,4,5-Trimethyl-4-heptanol	61.46 ± 3.11
Isobutyl acetate	44.99 ± 2.96	2-Ethyl-1-hexanol	51.28 ± 3.96
Ethyl dodecanoate	42.01 ± 1.54	1-Butanol	15.56 ± 0.91
Ethyl phenylacetate	29.05 ± 0.26	1-Propanol	11.62 ± 0.52
n-propyl acetate	25.29 ± 0.78	1-Decanol	8.38 ± 0.33
Isobutyl noctanoate	15.19 ± 0.33	(R)-3,7-Dimethyl-6-octen-1-ol	5.61 ± 0.29
Hexyl acetate	11.76 ± 0.12	2-Heptanol	5.22 ± 0.14
2-Methylhexyl butyrate	11.65 ± 0.23	(Z)-3-Hexen-1-ol	3.12 ± 0.09
2-Hydroxy-4-methyl-ethyl valerate	11.37 ± 0.15	2-Pentanol	2.27 ± 0.07
Isoamyl octanoate	10.95 ± 0.18	Pentyl alcohol	1.97 ± 0.09
Ethyl butyrate	7.59 ± 0.24	2-Methyl-4-penten-1-ol	0.74 ± 0.06
2-Methylbutyl phenylacetate	5.55 ± 0.15	Acid	
3-Methylbutyl methoxyacetate	5.04 ± 0.20	Acetic acid	515.86 ± 11.67
Butyl acetate	4.57 ± 0.36	Caproic acid	124.21 ± 6.88
Ethyl acetate	4.49 ± 0.06	Octanoic acid	52.73 ± 2.49
Ethyl propionate	4.22 ± 0.19	2-Hydroxy-tetradecanoic acid	17.48 ± 0.32
2-Hydroxy-4-methyl-ethyl valerate	4.15 ± 0.11	Isobutyric acid	14.68 ± 0.13
Ethyl dodecanoate	4.09 ± 0.21	Orthodecanoic acid	4.44 ± 0.23
2-Methylpropyl phenylacetate	3.91 ± 0.07	Others	
Ethyl heptanoate	3.87 ± 0.04	Eugenol	8.26 ± 0.16
Ethyl 3-hydroxy-4-(benzyloxy)butyrate	3.25 ± 0.05	à-Farnesene	7.82 ± 0.25
2-Methylpropyl caproate	2.96 ± 0.03	Acetoin	3.98 ± 0.11
Methyl acetate	2.45 ± 0.04	2-Methyldecane	2.87 ± 0.03
Ethyl (Z)-4-decenoate	2.43 ± 0.02	3-(Methoxymethoxy)-1-octene	2.85 ± 0.08
Propyl octanoate	2.28 ± 0.03	Hexadecane	2.82 ± 0.13
4-Chloro-3-hydroxy-3-methylbutyl ester	2.22 ± 0.01	6-Methyl-hexadecane	1.12 ± 0.09
Ethyl 3-hydroxyoctanoate	2.15 ± 0.11	3-Octanone	0.85 ± 0.02
Methyl (E)-10-octadecan-8-methacrylate	2.06 ± 0.09	(Z)-3-Methyl-2-decene,	0.69 ± 0.02
3-Methylbutyl methoxyacetate	1.94 ± 0.01		
2,6-Octadienoic acid, 3,7-dimethylmethyl ester	1.26 ± 0.05		

## Data Availability

Data are contained within the article.
